# ﻿Two new wolf-spider species in the genus *Hippasosa* from Asia (Araneae, Lycosidae)

**DOI:** 10.3897/zookeys.1226.137240

**Published:** 2025-02-10

**Authors:** Lu-Yu Wang, Muhammad Irfan, Zhi-Sheng Zhang

**Affiliations:** 1 Key Laboratory of Eco-environments in Three Gorges Reservoir Region (Ministry of Education), School of Life Sciences, Southwest University, Chongqing 400715, China Southwest University Chongqing China; 2 College of Plant Protection, Southwest University, Chongqing 400715, China Southwest University Chongqing China

**Keywords:** China, description, morphology, taxonomy, Thailand

## Abstract

Two new species of the wolf-spider genus *Hippasosa* Roewer, 1960 are described from Asia: *Hippasosathailandica***sp. nov.** (♂) from Thailand and *H.yunnanensis***sp. nov.** (♂♀) from China. Detailed species descriptions, morphological photographs, genitalia illustrations, and distribution maps of the new species are presented. Photographs of *H.qiongzhongensis* (Yin & Peng, 1997) are also provided for comparison purposes.

## ﻿Introduction

*Hippasosa* Roewer, 1960 is a genus of large wolf spiders that inhabit the banks of rivers or lakes (Jocqué et al. 2017), where they weave funnel-shaped webs like *Hippasa* Simon, 1885. The genus currently comprises 11 species (WSC 2024), with seven species occurring in the Afrotropical region—*Hippasosadewinterae* (Alderweireldt, 1996), *H.discrepans* (Roewer, 1960), *H.fera* (Strand, 1908), *H.ghost* (Jocqué & Jocqué, 2017), *H.grandis* (Alderweireldt, 1996), *H.guttata* (Karsch, 1878), and *H.pelliona* (Audouin, 1826). Two species were found in South Asia (*H.kumari* (Dyal, 1935), and *H.lanca* (Karsch, 1879)—and only one species, *H.qiongzhongensis* (Yin & Peng, 1997) recorded from China (Wang et al. 2021). *Hippasosapilosa* Roewer, 1960 is distributed across both Africa and Asia (Alderweireldt 1996; Sankaran and Caleb 2023). To clarify historical synonymies and address taxonomic inaccuracies, Sherwood (2022) provided a comprehensive revision of the taxonomic status of *Ocyale* Audouin, 1826 and *Hippasosa* genera, genera within Pisauridae Simon, 1890 and related Lycosidae Sundevall, 1833 families. The genera *Ocyale* and *Hippasosa* were previously considered synonyms. However, since the type species of *Ocyale*, *O.atalanta* Audouin, 1826, exhibits characteristics of Pisauridae and is regarded as a nomen dubium, Sherwood (2022) reinstated the genus *Hippasosa*. Most species formerly assigned to *Ocyale* were transferred to *Hippasosa*, while *Ocyale*, along with its type species *O.atalanta*, was treated as nomina dubia within Pisauridae.

While examining the Lycosidae specimens from China and Thailand we found two new *Hippasosa* species, which are described here: *H.thailandica* sp. nov. and *H.yunnanensis* sp. nov.

## ﻿Materials and methods

**Figure 1.**
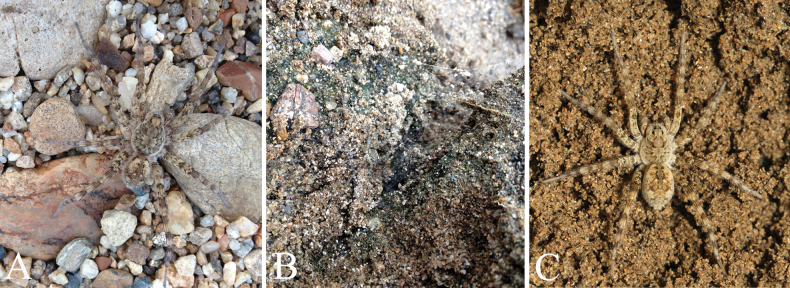
Photos of living specimens of the new species of *Hippasosa***A***H.thailandica* sp. nov., male **B***H.thailandica* sp. nov., web **C***H.yunnanensis* sp. nov. (female).

Photographs of living specimens were taken using an iPhone 4s (Fig. 1A, B) or a Canon EOS 7D camera with EF 100 mm F2.8L lens (Fig. 1C). All specimens were preserved in 75% ethanol and subsequently examined, illustrated, photographed, and measured with a Leica M205A stereomicroscope equipped with a drawing tube, Leica DFC450 camera, and LAS software (v. 4.6). Male pedipalps and epigynes were examined and illustrated after dissection. Female genitalia were cleared in pancreatin (Álvarez-Padilla and Hormiga 2007). Eye sizes were measured as maximum dorsal diameter. Leg measurements are presented as: total length (femur, patella and tibia, metatarsus, tarsus). The distribution map was created using ArcMap v. 10.2 (ESRI 2024) and further modified using Adobe Photoshop 2020 (Fig. 7). All measurements are given in millimeters. The specimens examined here are deposited in the spider collection of the School of Life Sciences, Southwest University, Chongqing, China (**SWUC**). Terminology follows Wang et al. (2015). Abbreviations used in the text: **ALE**, anterior lateral eye; **AME**, anterior median eye; **PLE**, posterior lateral eye; **PME**, posterior median eye.

## ﻿Taxonomy

**Family Lycosidae Sundevall, 1833**

### ﻿Genus Hippasosa

Taxon classificationAnimaliaAraneaeLycosidae

Roewer, 1960

B3BF8C2C-613A-5CC2-BB5B-661CE002CBB3

#### Type species.

*Hippasosapilosa* Roewer, 1960; gender feminine.

#### Diagnosis.

**Figure 2.**
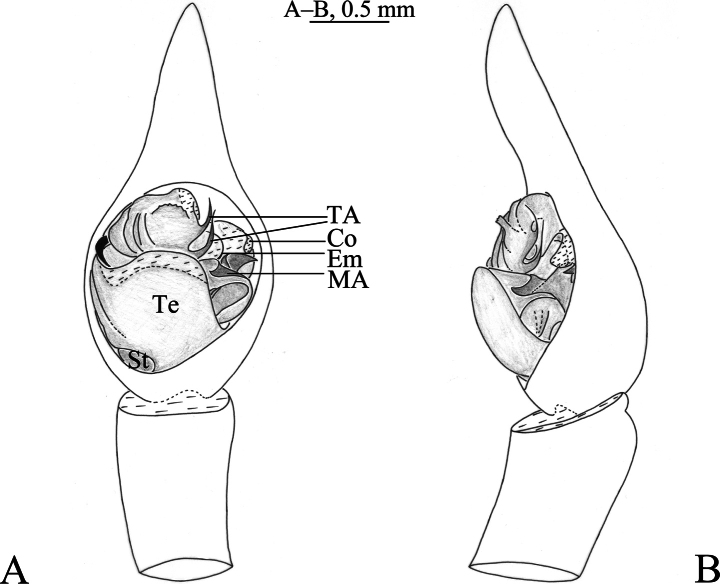
*Hippasosathailandica* sp. nov. holotype male **A** left male pedipalp, ventral view **B** same, retrolateral view. Abbreviations: Co = conductor; Em = embolus; MA = median apophysis; St = subtegulum; TA = terminal apophysis; Te = tegulum.

**Figure 3.**
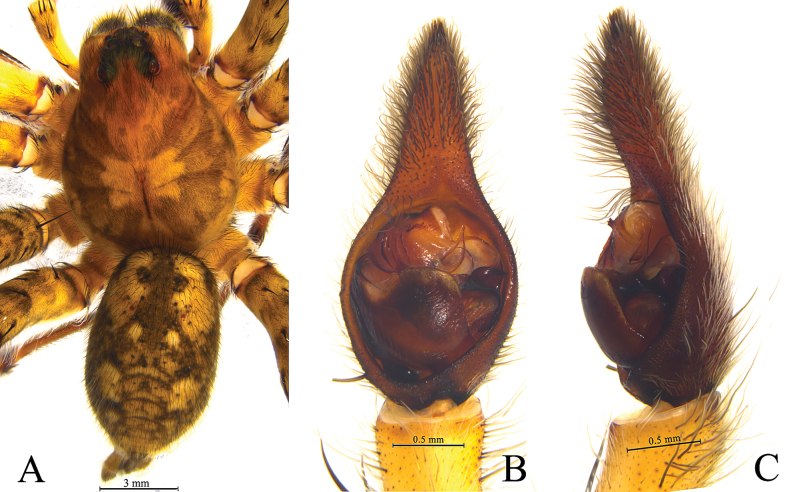
*Hippasosathailandica* sp. nov. holotype male **A** male habitus, dorsal view **B** left male palp, ventral view **C** same, retrolateral view.

**Figure 4.**
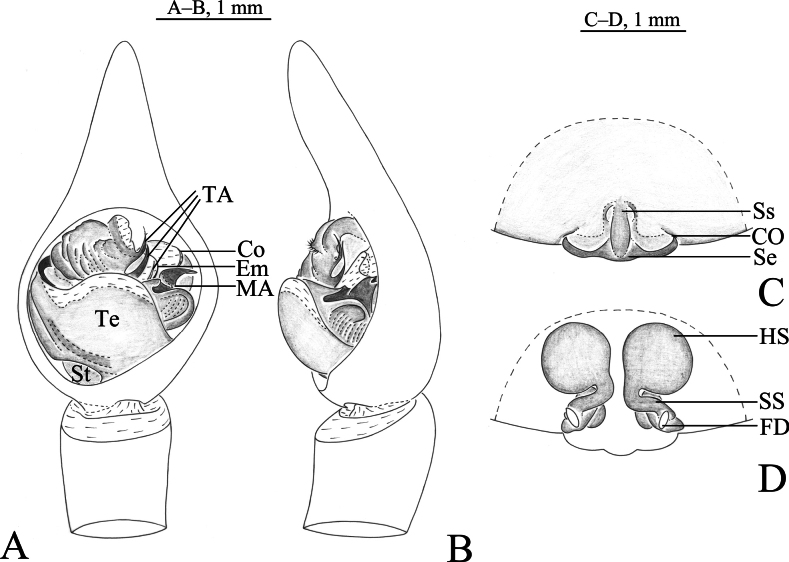
*Hippasosayunnanensis* sp. nov. **A, B** holotype male **C, D** paratype female **A** left male palp, ventral view **B** same, retrolateral view **C** epigyne, ventral view **D** vulva, dorsal view. Abbreviations: Co = conductor; CO = copulatory opening; Em = embolus; FD = fertilization duct; HS = head of spermatheca; MA = median apophysis; Se = septum; Ss = stem of septum; SS = stalk of spermatheca; St = subtegulum; TA = terminal apophysis; Te = tegulum.

**Figure 5.**
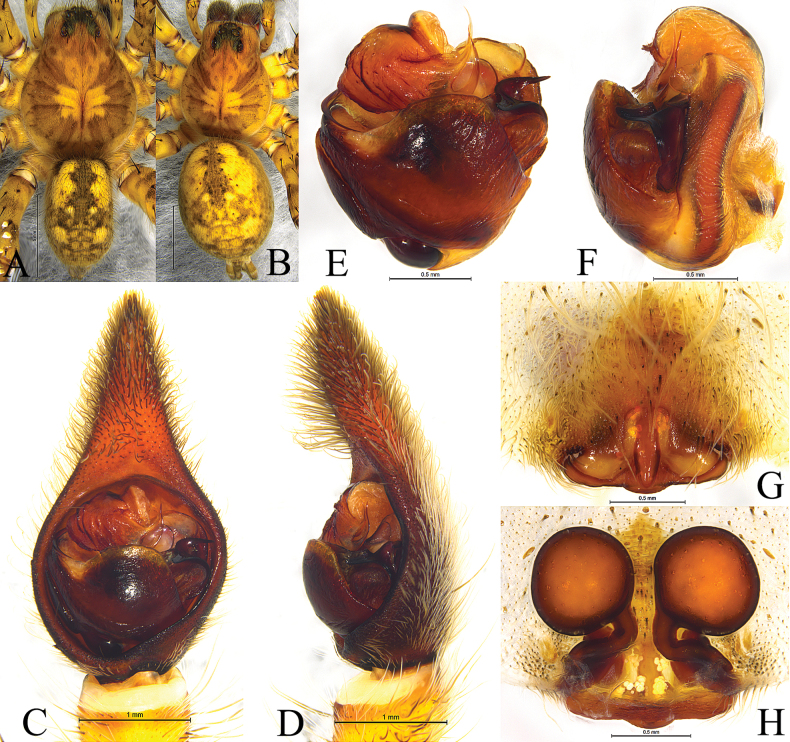
*Hippasosayunnanensis* sp. nov. **A, C–F** holotype male **B, G, H** paratype female **A** male habitus, dorsal view **B** female habitus, dorsal view **C** left male palp, bulb, ventral view **D** same, retrolateral view **E** left male palp, ventral view **F** same, retrolateral view **G** epigyne, ventral view **H** same, dorsal view.

**Figure 6.**
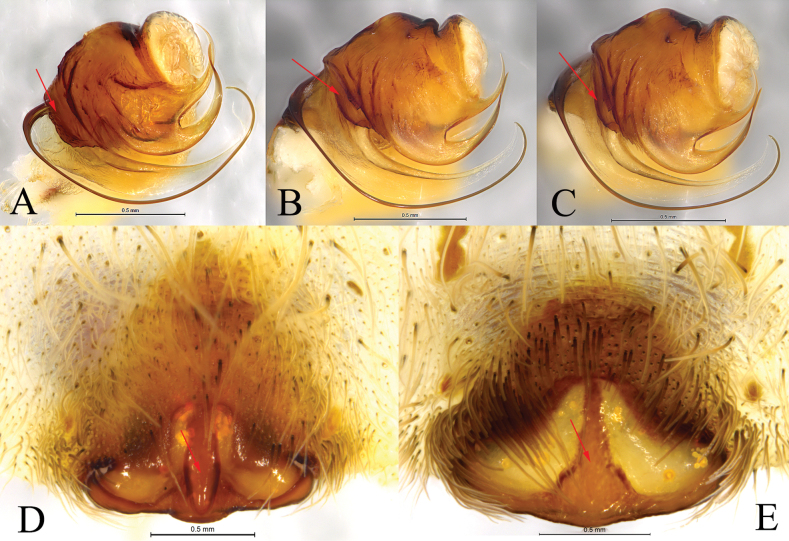
**A–C** embolus division **D, E** epigyne, ventral view. **A, D***Hippasosayunnanensis* sp. nov., paratype male **B, C, E***Hippasosaqiongzhongensis* (Yin & Peng, 1997) **A–C** arrow indicating the shape of prolateral edge of palea **D, E** arrow indicating the shape of septum.

This genus resembles *Hippasa* Simon, 1885 in weaving funnel-shaped webs (Wang et al. 2015: fig. 1B), male palps with a hook-like terminal apophysis, a slender embolus, and a bifid median apophysis (Figs 1A, B, 3B, C, 4A, B, 5C–F, 6A–C), and an epigyne covered with white setae (Figs 5G, 6D, E). *Hippasosa* can be distinguished from *Hippasa* by the following characters: body fatter (Figs 1, 3A, 5A, B, vs body slenderer, Wang et al. 2015: figs 4A, B, 6A, B, 8A, B), terminal apophysis three (Figs 6A–C, vs one, Wang et al. 2015: figs 3A, B, 4C, D), anterior arm of median apophysis as long as posterior arm (Figs 2A, B, 3B, C, 4A, B, 5C–F, vs longer than posterior arm, Wang et al. 2015: figs 3A, B, 4C, D), stem anchor-shaped (Figs 4C, 5G, 6D, E vs a strong, posteriorly extended scape, Wang et al. 2015: figs 3C, D, 4E, G, 5C, D, 6F, G, or with an obviously epigynal atrium, Wang et al. 2015: figs 7C, 8E, F), diameter of spermathecal head almost six times width of spermathecal stalk (Figs 4D, 5H vs 1.5–3 times, Wang et al. 2015: figs 3D, 4G, 5D, 6G, 7D, 8G).

#### Composition and distribution.

Eleven species known from Africa, Arabian Peninsula, and Asia.

### ﻿ Hippasosa thailandica sp. nov.

Taxon classificationAnimaliaAraneaeLycosidae

6D5B08AF-BDD0-59A0-9CB9-799FD303AE2C

https://zoobank.org/7DA08880-4CC1-4734-8DA3-DDCB0C05B202

**Figure 7.**
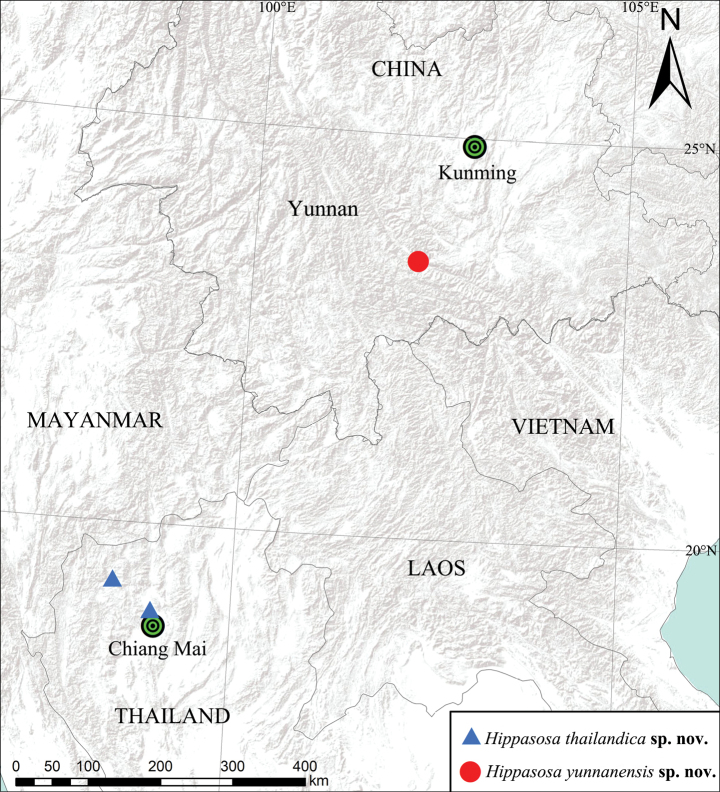
Distribution of *Hippasosathailandica* sp. nov. and *Hippasosayunnanensis* sp. nov. in Thailand and China, respectively.

Figs 1A, B , 2A, B , 3A–C , 7

#### Type material.

***Holotype*** • ♂, Thailand, Pai, 19°17.800'N, 98°27.925'E, elev. 478 m, 7 July 2014, Z.S. Zhang, L.Y. Wang, Z. Cao, X.K. Jiang and T. Lu leg.; SWUC-T-LY-20-01. ***Paratype***: • 1♂, Soi Pracha Nimit Bridge Tambon San Pong, Amphoe Mae Rim, Chiang Mai, 18°56.627'N, 98°58.518'E, elev. 318 m, 11 July 2014, Z.S. Zhang, L.Y. Wang, Z. Cao, X.K. Jiang & T. Lu leg.; SWUC-T-LY-20-02.

#### Etymology.

The species is named for the country where the type locality is located.

#### Diagnosis.

Males of *Hippasosathailandica* sp. nov. resemble those of *H.qiongzhongensis* and *H.yunnanensis* sp. nov. in having a similar body and palpal conformation (Figs 2–6; Wang et al. 2021: fig. 33), but the new species differs from them in having a curved retrolateral arm of the median apophysis (Figs 2A, 3B, C) vs strongly spiral in *H.qiongzhongensis* (Wang et al. 2021, figs 33C–F) and slightly spiral in *H.yunnanensis* sp. nov. (Figs 4A, B, 5C, D).

#### Description.

**Male** (holotype, Fig. 3A). Total length 16.91. Prosoma 9.35 long, 6.88 wide; opisthosoma 8.45 long, 5.15 wide. Carapace yellowish brown. Eye region black. Fovea longitudinal. Cervical groove and radial furrows indistinct. Eye sizes and interdistances: AME 0.43, ALE 0.28, PME 0.70, PLE 0.66; AME–AME 0.22, AME–ALE 0.10, PME–PME 0.48, PME–PLE 0.51. Clypeus height 0.90. Chelicerae elongate, blackish brown, with two promarginal and three retromarginal teeth. Labium and endites yellowish brown, longer than wide. Sternum yellowish brown, scutellate, with brown setae. Legs yellowish brown, with black pigmentation. Leg measurements: I 26.94 (7.11, 9.03, 6.30, 4.50); II 26.91 (7.37, 8.50, 6.55, 4.49); III 28.38 (7.13, 9.42, 7.30, 4.53); IV 33.42 (8.47, 10.45, 9.38, 5.12). Leg formula: 4312. Opisthosoma oval, dorsum yellowish brown, with black marking and lanceolate cardiac mark in anterior half. Venter yellowish brown.

***Palp*** (Figs 2A, B, 3B, C). Subtegulum situated at the base of the bulb prolaterally. Terminal apophysis with two branches, both hooked. Conductor broad, membranous. Median apophysis with two processes: anterior arm beak-like, retrolateral arm finger-like. Embolus arched, thin, and long.

**Female.** unknown.

#### Variation.

Males (*n = 2*): total length 16.91–18.79.

#### Distribution.

Thailand (Fig. 7).

### ﻿ Hippasosa yunnanensis sp. nov.

Taxon classificationAnimaliaAraneaeLycosidae

E16D5C18-C352-5453-A176-46DB36DE9DCB

https://zoobank.org/23868A53-F67B-4EC6-8B77-5C29127A1061

Figs 1C , 4A–D , 5A–H , 6A , 6D , 7

#### Type material.

***Holotype*** • ♂, China, Yunnan Province, Yuanjiang County, Lijiang Town, Nanhun Village, 23°26.502'N, 102°11.534'E, elev. 508 m, 13 June 2017, L.Y. Wang, R.B. Wu and Y.J. Lin leg.; SWUC-T-LY-21-01. ***Paratypes***: • 1♂ 2♀, with same data as holotype; SWUC-T-LY-21-02 to 04.

#### Etymology.

The species is named for the province in China where the type locality is located.

#### Diagnosis.

Males of *Hippasosayunnanensis* sp. nov. resemble those of *H.qiongzhongensis* in having a similar body and palpal conformation (Figs 4–6; Wang et al. 2021: fig. 33–35), but the new species differs from the latter in having the prolateral edge of the palea broad and rectangular (Figs 4A, 5C–F, 6A) vs small and crescent-shaped in *H.qiongzhongensis* (Fig. 6B, C) and the retrolateral arm of the median apophysis slightly spiral (Figs 4A, B, 5C–F) vs strongly spiral in *H.qiongzhongensis* (Wang et al. 2021: fig. 33C–F, 34A, B, 35A). The epigynal septum has a ridged stem (Figs 4C, 5G, 6D) vs depressed in *H.qiongzhongensis* (Fig. 6E).

#### Description.

**Male** (holotype, Fig. 5A). Total length 15.86. Prosoma 9.49 long, 7.11 wide; opisthosoma 6.65 long, 4.31 wide. Carapace yellowish brown. Eye region black. Fovea longitudinal. Cervical groove indistinct, radial furrows distinct. Eye sizes and interdistances: AME 0.45, ALE 0.31, PME 0.68, PLE 0.64; AME–AME 0.25, AME–ALE 0.12, PME–PME 0.55, PME–PLE 0.54. Clypeus height 0.35. Chelicerae elongate, blackish brown, with two promarginal and three retromarginal teeth. Labium and endites yellowish brown, longer than wide. Sternum yellowish brown, scutellate, with brown setae. Legs yellowish brown, with black pigmentation. Leg measurements: I 26.20 (7.61, 8.27, 6.18, 4.14); II 27.57 (7.46, 9.19, 6.62, 4.30); III 28.35 (7.67, 9.48, 7.02, 4.18); IV 33.59 (8.67, 10.90, 9.10, 4.92). Leg formula: 4321. Opisthosoma oval; dorsum yellowish brown, with black marking, and lanceolate cardiac mark in anterior half. Venter yellowish brown.

***Palp*** (Figs 4A, B, 5C–F, 6A). Subtegulum situated prolaterally at the base of the bulb. Terminal apophysis with three spiny branches; third branch membranous. Median apophysis sinuous, with two arms: anterior arm beak-shaped, retrolateral arm hooked, helical with a twisted tip. Conductor wide, membranous with rounded end. Embolus arched, thin, and long.

**Female** (Paratype, SWUC-T-LY-21-02, Fig. 5B). Total length 19.20. Prosoma 9.30 long, 7.24 wide; opisthosoma 9.91 long, 7.47 wide. Eye sizes and interdistances: AME 0.44, ALE 0.29, PME 0.77, PLE 0.69; AME–AME 0.27, AME–ALE 0.14, PME–PME 0.43, PME–PLE 0.53. Clypeus height 0.37. Leg measurements: I 26.20 (7.16, 9.20, 5.87, 3.97); II 27.10 (7.45, 9.35, 6.11, 4.19); III 27.77 (7.67, 9.18, 6.91, 4.01); IV 33.09 (8.50, 11.21, 8.72, 4.66). Leg formula: 4321. All other morphological characters as in male.

***Epigyne*** (Figs 4C, D, 5G, H, 6D). Posterior margin of septum wavy. Septum longer than wide, anchor-shaped, with a ridged stem. Spermathecal heads large, round. Spermathecal stalk short. Fertilization ducts short.

#### Variation.

Males (*n* = 2): total length 15.86–17.03, female (*n* = 2): total length 19.20–21.70.

#### Distribution.

Known only from the type locality, Yuanjiang County, Yunnan, China (Fig. 7).

## Supplementary Material

XML Treatment for Hippasosa

XML Treatment for Hippasosa thailandica

XML Treatment for Hippasosa yunnanensis

## ﻿Additional information

### Author ORCIDs

Lu-Yu Wang https://orcid.org/0000-0002-5250-3473

Muhammad Irfan https://orcid.org/0000-0003-0445-9612

Zhi-Sheng Zhang https://orcid.org/0000-0002-9304-1789

### Data availability

All of the data that support the findings of this study are available in the main text.
